# Stigmatization among people living with HIV in Hong Kong: A qualitative study

**DOI:** 10.1111/hex.12535

**Published:** 2017-02-14

**Authors:** Phoenix K. H. Mo, Charlson T. Y. Ng

**Affiliations:** ^1^ School of Public Health and Primary Care The Chinese University of Hong Kong Hong Kong Hong Kong

**Keywords:** Hong Kong, people living with HIV, qualitative, stigma

## Abstract

**Background:**

HIV/AIDS is one of the most stigmatized medical conditions across the world. Self‐stigma is prevalent among people living with HIV (PLHIV) and a major obstacle to HIV prevention and care.

**Objective:**

This study aimed to describe the experiences of stigmatization and explore the possible factors that might be associated with stigmatization among PLHIV in Hong Kong.

**Design:**

Qualitative in‐depth interviews were conducted.

**Setting and participants:**

15 PLHIV were recruited from two local non‐governmental organizations on HIV prevention.

**Main variables studied:**

Participants were interviewed about their views and feelings towards oneself as a PLHIV and contributing factors, experiences of discriminations, stigmatizing behaviours, issues about disclosure, social relationships and potential impact of HIV.

**Results and conclusions:**

Thematic analyses revealed three levels of factors which might be associated with stigmatization: (i) intrapersonal level (misconceptions about HIV, attribution of self‐responsibility, severe state of illness, side‐effects of medication), (ii) interpersonal level (discrimination, social rejection) and (iii) social level (mass media, public stereotypes). Findings provide important insights into which interventions to reduce stigmatization of PLHIV could be designed.

## 1 INTRODUCTION

HIV is a serious global epidemic causing heavy social and medical costs. Although global HIV prevalence has levelled off, the total number of people living with HIV (PLHIV) is increasing steadily due to the on‐going acquisition of HIV infection, longer survival times of PLHIV and a growing general population.^1^ In Hong Kong, the HIV prevalence was low among the general population but high among some specific populations (e.g. 5.9% among men who have sex with men).^2^ Among the new HIV cases reported in the second quarter of Hong Kong, 86.7% were male, and 35.7%, 36.9% and 4.5% were infected through heterosexual, homosexual and bisexual contact, respectively.^3^ The figures are comparable to those of the UK, which shows that out of those newly diagnosed with HIV in 2015, 75% were male, and 38.1% and 56.1% were infected through heterosexual and homosexual sex contact, respectively.^4^ Despite the promising efforts in counteracting HIV, HIV continues to be a major public health concern in Hong Kong.

HIV has long been regarded as one of the most stigmatized medical conditions.^5^ According to Goffman,^6^ stigma is defined as an attribute linking a person to a set of undesirable characteristics that may lead to prejudice and discrimination. While public stigma is the reaction that the general population has to the individuals who are considered different, self‐stigma occurs when “people with devalued status who internalize the discriminatory beliefs and experience diminished self‐esteem and self‐efficacy”.^7^ Corrigan suggested that both public stigma and self‐stigma comprise three components: stereotype (negative belief about the group/self), prejudice (agreement with belief and/or negative emotional reaction) and discrimination (behaviour response to prejudice).^8^ Self‐stigma has further been explained by the cognitive‐affective‐behavioural model.^9^ According to the model, the stigmatized individuals perceive their stigmatized identity as a burden and taint of their life, which lead to negative sense of self (self‐stigmatizing cognitions). Such conceptions might lead to a myriad of negative affective responses, encompassing feelings of anger, fear and shame (self‐stigmatizing affect) which, in turn, resulting in identity concealment, social avoidance and self‐denigration (self‐stigmatizing behaviours).

Despite the continuous intervention efforts in combating HIV stigma, public stigma towards PLHIV remains ubiquitous across the globe. In a meta‐analysis of 21 studies, public stigma towards HIV/AIDS is the greatest compared to genital herpes, hepatitis, drug abuse and cancer.^10^ The high level of stigma towards PLHIV can be due to several causes. First, a high level of self‐responsibility is attached to HIV as the acquisition of HIV is due to behaviours that can be preventable, such as unsafe sex and needle sharing.^11^, ^12^ Some behaviours associated with HIV, such as same‐sex behaviours, are also considered immoral and are thus condemned by the society.^5^ Misconceptions about HIV transmission routes and overestimation of the perceived contagiousness and risks through casual contact further evoke stigmatization towards HIV.^12^ These cause an intensive level of blame and disapproval against PLHIV, resulting in social rejection, discrimination and prejudice.^13^, ^14^

It is contended that stigma against PLHIV is even more intense in the Chinese context, as the behaviours related to HIV infection, such as same‐sex behaviours, multiple sex partnership and injecting drug use, are generally perceived as defying acceptable social norms and culture.^15^, ^16^, ^17^ High level of ignorance, misconception and fear of HIV also contribute to the high level of stigma towards PLHIV in the Chinese context.^18^, ^19^ Furthermore, face concern, which refers to social image and social worth that are garnered based on one's performance in an interpersonal context, is particularly important in the Chinese society.^20^, ^21^ Yang and Kleinman^22^ proposed that while the concept of “face” represents social power, capital and a person's value in society, PLHIV experiences loss of face as a result of violation to cultural norms, which greatly affects their access to social capital and closely parallels how stigma works in Chinese society. In Hong Kong, studies have documented a high level of unfavourable attitudes and stigmatizing behaviours towards PLHIV. For example, a population‐based study among Hong Kong adults reported that nearly half of them exhibited discriminatory attitudes towards PLHIV.^23^ A comparative study on the public's attitude towards different types of infectious diseases have shown that the level of public stigma towards HIV was the highest compared to other infectious diseases such as severe acute respiratory syndrome (SARS) and tuberculosis (TB).^24^ PLHIV are also disadvantaged in terms of economic and social opportunities. For example, a local study reported that 20% of the studied companies would dismiss an employee if he or she was HIV+, and only a few companies indicated that they would provide counselling and support to an HIV+ employee.^25^ Comparative study on employer attitudes towards PLHIV in Beijing, Hong Kong and Chicago revealed the trend of reluctance to hire PLHIV are most pronounced among employers from Beijing and Hong Kong.^26^ Study among PLHIV in Hong Kong has also shown that over 50% felt that they were discriminated in different settings such as in the workplace and in social relationships.^27^

HIV stigma poses a significant impediment to public health across the globe and a key obstacle to HIV treatment, prevention, care and support.^28^, ^29^ It is noticeable that many PLHIV have internalized the negative labels attached to them and experienced a high level of self‐stigma. In one study among 322 PLHIV in China, 78% of them had the feeling of negative worth and 58% of them were unwilling to disclose their HIV status.^30^ It is also reported that the level of internalized stigma was similar between PLHIV diagnosed less than 1 year ago and more than 1 year, indicating that level of self‐stigma did not reduce across time.^31^ Self‐stigma is significantly associated with various negative outcomes such as worse psychosocial well‐being,^32^ worse mental health^31^, ^33^, ^34^, ^35^ and worse physical health,^36^, ^37^ suicidal ideation,^38^ poorer access to health services,^35^, ^39^, ^40^ delay or non‐adherence to medication treatment,^41^, ^42^ and poor quality of life^43^, ^44^ among PLHIV.

Given the potential adverse consequences of HIV stigma, exploring the factors associated with stigma is important to understanding and ameliorating stigma among PLHIV. To date, most of the studies on stigma have focused on how self‐stigma might affect health and relatively little attention has been paid to the factors which may contribute to the perception of stigma among PLHIV. Studies of other health conditions indicated that the way people attribute their disease may influence their internalization of stigma. For example, local studies on people with schizophrenia and parents of children with autism showed that attribution of perceived controllability of the disease, personal responsibility to the cause of the illness and self‐blame was significantly associated with higher levels of self‐stigma.^45^, ^46^ However, one local study looking at factors associated with self‐stigmatization using the attribution model for PLHIV, including self‐blame, responsibility for contacting HIV and internal controllability for contracting HIV, showed that although attributions of control predicted attributions of responsibility which, in turn, predicted self‐blame, the linkage between self‐blame and self‐stigma was not significant.^47^ Other factors have also been proposed. Recent studies in adults living with HIV have found that optimism predicts lower levels of HIV‐related stigma indirectly through increasing psychological well‐being^32^ and also that personal meaning predicts lower level of HIV‐related stigma indirectly through increasing social support.^48^ These mechanisms have not been explored in the local context.

Furthermore, mass media has often been used to shape public attitudes and knowledge about HIV. It has been suggested that Chinese individuals are more likely to obtain HIV information from mass media than from interpersonal sources.^49^ A content analysis of articles about HIV published in Chinese newspapers over a decade revealed that individuals who contracted HIV through socially unacceptable means were devalued as non‐descript members of a deviant and dangerous group.^50^ However, from our understanding, how mass media might lead to self‐stigma among PLHIV in the Chinese context has not been examined.

There is an urgent need to understand how internalization of stigma is formed and intensified among PLHIV in the Chinese context so that tailored services and intervention can be provided to this population. The purpose of this study was to describe and explore the possible factors that may contribute to internalization of stigma among PLHIV in Hong Kong using a qualitative approach.

## 2 METHODS

### 2.1 Participants and procedures

Participants were adults living with HIV in Hong Kong. Inclusion criteria were (i) age of 18 or above, (ii) being diagnosed with HIV for more than 6 months, (iii) being able to speak in Cantonese which is the native language of Hong Kong and (iv) mentally competent in taking part in a 1.5‐hour in‐depth interview.

Participants were recruited from two local non‐governmental organizations (NGO) on HIV prevention using convenience sampling. Staff of the participating NGO identified their members who fulfilled the inclusion criteria, briefed and referred them to contact the research staff who would make an appointment to meet with them. At the meeting, participants were briefed about the purpose and logistic of the study again. Written informed consent was obtained before the in‐depth interview was administered by the research staff in a private room, in the absence of any third person. Data confidentiality was assured. A total of 15 eligible members were referred to the research team; all of them provided written consent to join the study and completed the in‐depth interview. Each interview lasted for about 1.5 hours. Ethical approval was obtained from The Chinese University of Hong Kong Survey and Behavioral Research Ethics Committee.

### 2.2 MEASURES

#### 2.2.1 Socio‐demographic and medical characteristics

Participants’ socio‐demographic characteristics, including age, gender, education level and employment status, were obtained. Medical characteristics, including length of diagnosis, disease stage, and most recent CD4 count, were also obtained.

#### 2.2.2 Interview guide

Interview questions were open‐ended and broad to elicit a detailed description of participants’ experiences. The protocol was developed by a group of panel consisting of researchers at the field of HIV, staff at the participating NGO and PLHIV. Topics of the questions included how their views towards oneself as a PLHIV and contributing factors, their feelings towards oneself as a PLHIV and contributing factors, experiences of discriminations, stigmatizing behaviours, issues about disclosure, social relationships and potential impact of HIV.

### 2.3 Data analysis

The interviews were audio‐recorded and transcribed verbatim. Responses characterizing the factors associated with internalization of stigma were noted and analysed inductively using thematic analysis. The coding process of thematic analysis involves recognizing patterns from the data and encoding them prior to the process of interpretation.^51^, ^52^ The responses were read and reread several times, across both questions and respondents, to increase familiarity with the data. Notes were made to reflect initial impressions and were progressively conceptualized into broader themes that best captured participants’ viewpoints.^53^ The coding framework and results were discussed between the authors, and any discrepancies were resolved to safeguard the reliability of the findings.

## 3 RESULTS

### 3.1 Participants

A total of 15 PLHIV completed the study. The majority of the participants were male (93.3%) and about two‐third of them were in the age of 45 or above (60%). One‐fifth (20%) of them had a post‐secondary level of education or above. Half of the participants (53.3%) were heterosexual. One‐third of the participants were infected with HIV through heterosexual contact with sex workers, and 26.7% were infected through homosexual contact. In terms of medical characteristics, the average length of time since diagnosis was 7.72 years (range <1 to 22 years). About half of them (46.7%) had a CD4 cell count of 500 uL or above. A majority of them (93.3%) reported having regular medical check‐up (Table 1). The socio‐demographic background of the participants is reflective of the HIV population in Hong Kong.

**Table 1:** Socio‐demographic and medical characteristics of participants (N=15)

	N (%)
Gender	
Male	14 (93.3)
Female	1 (6.67)
Age	
18‐25	1 (6.67)
26‐30	0 (0.00)
31‐45	5 (33.3)
46‐50	6 (40.0)
60+	3 (20.0)
Sexual orientation	
Heterosexual	8 (53.3)
Homosexual	6 (40.0)
Bisexual	1 (6.67)
Level of education	
Primary	2 (13.3)
Secondary	10 (66.7)
Post‐Secondary	3 (20.0)
Duration of HIV infection	M=7.72 years
Route of HIV transmission	
Heterosexual contact with sex workers	5 (33.3)
Heterosexual contact with partner	1 (6.7)
Heterosexual contact (sex worker or partner)	1 (6.7)
Homosexual contact	4 (26.7)
Not sure	4 (26.7)
Having regular medical check‐up	
Yes	14 (93.3)
No	0 (0.00)
Sometimes	1 (6.67)
Having AIDS‐related complications	10 (66.7)
Recent CD4 amount	
<200	2 (13.3)
>200	5 (33.3)
>500	7 (46.7)
Forgot	1 (6.67)

### 3.2 Factors contributing to self‐stigmatization among PLHIV

Findings from the interviews seem to suggest that various levels of factors contribute to self‐stigma among PLHIV. In particular, participants’ responses to the open‐ended questions were conceptualized in the following themes:

#### 3.2.1 Intrapersonal level

##### Misconceptions about HIV

Some members described that misconceptions about HIV contributed to their self‐stigmatization. Misconceptions about HIV seemed to be common among the members. Such misconceptions included misunderstandings in HIV‐related treatment and complications, longevity after HIV infection, and its mode of transmission. For instance, members perceived that HIV was equal to death and no effective treatment was available. These misconceptions have brought them feelings of fear and hopelessness about the future, such negative feelings about HIV further lead to a devaluation of oneself. For example, one participant reported:

I had no idea about HIV. Um… It should be an incurable disease, which was the only thing I knew. Therefore, I was really afraid of being identified.<Participant 11>

Similarly, other participants described their misconception that HIV could also be transmitted by casual contact, such as sharing dishes and mosquito bites. They hence isolated themselves or avoided interacting with others in order to avoid transmitting the disease to others:

I feared that the virus might be transmitted to my wife. At that moment, I was really worried when dinning with my family members. Indeed, I was even afraid that mosquito could transmit HIV from my body to them. Therefore, I seldom went out to meet the others.<Participant 7>

##### Attribution of self‐responsibility

HIV infection has been considered as an illness that is attributed to behaviours that are preventable and controllable. For those members whose acquisition of HIV was due to unsafe sex, they deemed that it was their own fault and responsibility, and they showed feelings of devalued self‐worth. Along with being ashamed of their infection, they also revealed that they would feel embarrassed and guilty if their serostatus was disclosed.

I felt mad about myself when I was diagnosed with HIV. I really hate myself … Since I had unprotected sex with a female sex worker in mainland China, I was really afraid that people around me would know how I got HIV.<Participant 6>

In particular, several informants pointed out that HIV tainted their life because they contracted HIV due to behaviours which were considered immoral:

Participant: ……. HIV is a taint of my life!

Researcher: Could you explain a bit more why?

Participant: Taint? Hey, brother! Why not? Your body did not have that virus before, but you contracted HIV because you have patronized sex workers. If you suffer from HIV, your body is no longer holy<Participant 9>

These members revealed that contracting HIV was totally their personal controllability and responsibility. They happened to show high level of self‐blame, which, together with negative socially view of HIV constructed in Hong Kong, developed self‐stigmatizing cognitions and feelings towards themselves.

##### Severe state of illness

Findings of the study also suggest that complications of HIV not only bring insidious effects to participants’ physical health, they also undermine their psychological and mental well‐being, leading to isolation and a negative sense of self:

My physical appearance has become really strange after acquiring this disease. My skin was full of lesions and ulcers. I looked very horrible at that time, but I still wanted to do Tai Chi. As I wanted to avoid someone asking me what disease I have suffered from, I went to the park, where no one recognized me, and did Tai Chi myself. <Participant 2>

I weighed only 70 pounds at that time. My physical appearance was far beyond my real age. If a person's weight suddenly drops, people around you will suspect that you have got HIV. Thus, I wore long‐sleeves shirt even in really hot weather, in order to hide my body shape. <Participant 5>

My immunity was extremely low at this time, and I could not even leave from the bed. As I was also infected with Candida, my physical health was frail. I was just like a disable person who needed to rest all the time. During hospitalization, my family members needed to look after me every morning. I had the feeling that HIV infection was a burden of my life, but it has shifted to my parents. I felt guilty of my mistake.<Participant 14>

As a result of disease progression as well as other symptoms, such as weight loss and skin lesion, became evident, their extent of self‐stigma appeared to become signified. These altered their body images and made them devaluated themselves. In the eyes of the members, these sudden changes could be seen as an evidence of their HIV infection. To avoid being suspected as PLHIV, they intended to hide those symptoms or withdraw themselves from their social network.

##### Side‐effects of medication

Thus far, HARRT has been proven to be effective in keeping the viral load of PLHIV undetected. Having high level of compliance, their longevity would be prolonged. Despite the minimal side‐effects in majority of PLHIV, still, a few have unusual responses after taking HARRT. These unpleasant side‐effects have created enormous psychological and physical sufferings on their life. Some of them noted that they did not commit into their social life and they tended to have a negative evaluation of themselves because of the side‐effects of the medication.

Due to the side effects of medication, I have cried and stood near the window. I wanted to throw out all the drugs and end my life. This medicine is like a grenade and I need to bear it all the time. When would it set off? I really don't know. Such unpleasant side effects have bothered my social life. I am so used to feeling dizzy, losing appetite and going to washroom just after taking this. At present, I will not dine out with my friends, as it is not pleasant to ask them to wait for me all the time.<Participant 12>

#### 3.2.2 Interpersonal level

##### Discrimination

Discrimination seems to be a major factor causing the stigmatization among PLHIV. Findings from the study reveal that many of the participants had the experiences of being discriminated, and such experiences took place in various contexts. Surprisingly, some PLHIV reported experiencing discrimination in clinic setting, which was mainly manifested by the poor attitudes of medical professionals, and unusual arrangement of consultations and nursing care. One member reported his experience:

…The hospital assistant put my tableware separately. It seemed that she intended to separate my tableware from others. But…never mind… I dared not to argue with them. Um… I remember once I have been hospitalized for nearly two months but the doctor did not prescribe me any medication. I asked the doctor ‘Do I need any medication?’ He felt strange and did not answer me. After a few days, I asked him again and he replied me loudly ‘Do you know what disease you suffered? It is AIDS!!’ His attitude made me think that I was convicted of any misbehavior. As there were other patients here, I thought they might hear what the doctor said. I felt embarrassed and dismal. <Participant 3>

Poor perception of self as a PLHIV seems to build from such discriminatory experiences. When PLHIV find that they do not receive any respect and support from others, they are more likely to conceal their identity and show negative feelings towards themselves. The negative perception might be further exacerbated when it comes to the clinic setting, as clinic staff is expected to show empathetic understanding to every patient.

##### Social rejection

For most of the participants, HIV infection is considered as a shame to both PLHIV and the groups they belong to, from the interview it was ubiquitous that many members were rejected from their social circle.

The moment my parents recognized my infection status, my mother kept on murmuring and blaming me, whereas my father said nothing. They did not accept me at all, so I left my home eventually… I hate myself.<Participant 8>

Because of my infection, I divorced with my wife. Actually, our marriage lasted for 10 years after my infection, but we did not have sex during that time. She was really afraid of me. I later realized she had extramarital relations and we eventually broke up. I could not accept that…I was really upset and I have tried to commit suicide… It seemed that everyone would not respect me and like me…, which also made me dislike myself.<Participant 6>

Findings suggest that participants seem to be rejected from their significant others, and these unpleasant responses tend to be the prime factor for their self‐stigmatization. Social rejection is particularly deleterious that PLHIV might think that their identity incur shame to them and their families as well. It also appears to make them believe that being PLHIV would only receive aversive reactions from their social network, leading to a negative perception towards themselves.

#### 3.2.3 Societal level

##### Public Stereotypes

Findings also suggest that public stereotype about HIV appears to be the cause of their self‐stigmatization. Some respondents revealed that PLHIV were perceived by the society as “Lan Gwan” (someone who always patronizes sex workers) and “Dai Sei”(someone who deserves to die), these stereotypes have further portrayed PLHIV as “bad people,” which jeopardizes their level of self‐worth:

From the perspective of society, PLHIV are “Dai Sei” as they venture their lives to get sexual pleasure. You could have chosen to use condom, but why hadn't you used that? To them, All PLHIV are regarded as “Lan Gwan”, drug users or “Tong Zhi” (homosexuals). However, it is understandable, as I also had the same belief with them.<Participant 10>

These public stereotypes constitute a negative impression towards PLHIV, constructing devaluated and marginalized social status on them. PLHIV are highly aware of the stereotype attached on them, and they would then internalize those stereotypes and prejudices onto themselves. The stereotypes imbued by the society are that PLHIV should be drugs users, homosexuals or promiscuous, which are bounded to be negative.

##### Mass Media

The mass media, mainly the TV, advertisements and newspapers, serves as a tool to disseminate message and constructs a norm within the society. Most of the respondents seem to blame the media for being the main source of creating unwelcoming and hostile climate towards PLHIV. As one member explained:

…Media seemingly only focuses on the dark side of PLHIV. For example, newspapers usually use an exaggerated title to describe a PLHIV who has committed suicide. It has instilled the concept into general public that all PLHIV will do the same thing as well.

Members claimed that the media was prone to cast a negative light on them by developing negative portrayal of PLHIV:

In some movies or TV programme, it was common to see that the actor would curse their enemy to die of HIV. Although it seemed to be to a joke, it might defame us. <Participant 3>

I watched one documentary from TV, showing a group of PLHIV in Africa. It appeared that all of them would die soon, which made people think that HIV was like a Hung Shui Mang Shau (Dreadful monster) and that all people should escape from them. If I were not PLHIV, I would also have been scared by this documentary. Actually, by taking medicine regularly, we would never progress to this stage ……<Participant 12>

The negative representations of PLHIV by the media have built an adverse image towards PLHIV, some members appear to be affected by such portrayal and showed negative feelings towards themselves:

I firmly remember one TV advertisement produced by the government, describing a fatal pyramid of AIDS. The major message was that HIV was equivalent to death, drug users, gay man and promiscuity. This message, eventually, has rooted into everyone's mind. The moment I knew my infection status, I could only imagine that fearful advertisement and I strongly believed that I would be dead soon. My life was blue and dull…<Participant 5>

## 4 DISCUSSION

The present study was the first attempt in understanding the stigmatizing experiences and the possible factors that may be related to self‐stigmatization among PLHIV in Hong Kong.. Findings show that participants face strong stigmatization in various ways and suggest a number of factors that may be associated with their self‐stigmatization. First, from the intrapersonal level, attribution of responsibility to self seems to be associated with self‐stigmatization. Our findings suggest that many participants have shown feelings of shame and guilt as they considered themselves responsible for the infection. As most of the participants acquired HIV through heterosexual contact with sex workers or homosexual contact with men, it is conceivable that participants demonstrated an internal attribution of the disease and thus reporting negative feelings of shame and guilt and a high level of self‐stigmatization. Findings are consistent with a local study which shows that the Hong Kong general public perceived PLHIV as more responsible and blameworthy of their diseases than other conditions such as SARS and TB.^24^

Findings also suggest that physical challenges, such as severe HIV symptoms or medication side‐effects; tend to increase their level of self‐stigmatization. Participants described how they wanted to isolate themselves because of the physical symptoms, and how their physical symptoms made them felt negative about themselves. Previous work has suggested that stigma associated with HIV increases as symptoms become more apparent to others.^11^ Findings are also consistent with previous studies that PLHIV who greater severity of HIV symptoms experienced higher levels of self‐stigma.^5^ As HIV is a concealable condition, the appearance of physical symptoms might be one of the primary cues that someone is infected with HIV. The intense physical sufferings attached to HIV may cause PLHIV distress and frustration.

Findings of the present study also reveal that misconception about HIV is another factor leading to self‐stigmatization. As suggested by the data, misconceptions about HIV, even among PLHIV, were highly prevalent. Findings corroborate with local studies that people in Hong Kong have poor knowledge about HIV.^23^ It also supports previous studies that show the pathway between lack of knowledge about HIV to increased felt stigma and decreased intention to disclose one's HIV status.^54^ Despite the critics that education alone might not be effective in reducing stigma,^55^ the present study suggests that providing PLHIV knowledge of the disease is still the key and first step in stigma reduction.

In the interpersonal level, findings also suggest that discrimination and social rejection emerged as a factor driving self‐stigmatization. The present study indicates that PLHIV experience discrimination and social rejection in various settings. In the study, most of the participants gave detailed accounts on their feelings of social isolation or experiences of being rejected by the family and society. Some of them even reported facing discrimination in the health‐care settings. Indeed, stigma attached to HIV remains ubiquitous in Hong Kong.^23^ Study in China also revealed that people hold stigmatizing attitude to PLHIV as they are blamed for their acquisition of the disease.^19^ Facing both social rejection and discrimination experiences, PLHIV may endorse their stigmatizing attitudes and internalize negative feelings into themselves, which, in turn, develop poor sense of self and self‐esteem.

At the society level, the media also plays an important role in shaping the self‐stigma of PLHIV, mainly through delivering biased messages about HIV and developing a negative representation of PLHIV. Participants described how the stereotypes of PLHIV as promiscuous, homosexuals and drug abuses created by the media has caused them frustration and discouragement. As the HIV population in Hong Kong are predominantly male who contract HIV through sexual behaviours, it may further reinforce the public idea that only those male who are homosexuals or promiscuous would get HIV, creating further stigmatization. The negative connotations carried by HIV are consistent with a local study that a sizeable proportion of the respondents hold negative perceptions about PLHIV.^23^ Such misrepresentation of HIV tend to be partly due to the inaccurate and biased information presented by the media in the early 1990s, which framed HIV negatively as a dead pyramid, indicating that HIV was a fatal illness with no treatment available. Despite that fact that the disease has been transformed into chronic illness in the post‐HAART era, the message has not been well disseminated to the public. The negative perceptions and stereotypes endorsed by the society might intensify the beliefs of PLHIV that they are inferior and should be blamed and stigmatized by the society. Results support the extant literature that public and self‐stigma are inextricably associated,^56^ and PLHIV may be at risk for internalizing the society's negative views towards themselves and holding a stigmatizing views towards themselves as a result.

HIV‐related stigma has long been regarded as a hindrance to public health effort in controlling HIV, therefore interventions reducing stigma among PLHIV are highly warranted. Our findings suggest various ways in which HIV stigma could be reduced. First, the present findings reveal that misconceptions about HIV could intensity stigmatization; therefore, there is a need for both PLHIV and the public to be better educated about HIV and this should be given the highest priority in HIV stigma reduction. Local studies have revealed that misconceptions about HIV in Hong Kong fall particularly on its mode of transmission and mortality rate.^23^ Therefore, education in these two particular areas of HIV should be introduced in both clinic and community settings. Other studies also suggest that interventions designed to address negative self‐image or stigma of HIV could include a variety of modes such as individual or counselling, cognitive behavioural therapy, social support and empowerment.^57^, ^58^ In particular, family of the PLHIV and health‐care professionals should also be targeted in interventions as the present study shows that discrimination and social rejections also originated from them. There has been evidence that community‐based HIV stigma reduction programme was effective to help family members gain a richer understanding of HIV stigma, ways to cope with it and ways to be more supportive to PLHIV.^59^ To reduce HIV stigma in the health‐care setting, interventions must focus on the individual, environmental and policy levels.^60^ At the individual level, it is important to increase awareness of stigma and the benefits of reducing stigma among health workers. In the environmental level, there is a need to ensure that health workers have the information, supplies and equipment necessary to practice universal precautions and prevent transmission of HIV. It is also important to enact policies that protect the safety and health of patients and health‐care professionals in order to prevent discrimination against PLHIV. However, this might be difficult to achieve given the lack of policies to prevent discriminations against vulnerable groups in Hong Kong.

The study also indicate that mass media is the main culprit of creating a biased image for PLHIV, thus leading to their stigmatization. Interventions to reduce HIV stigma should therefore extend beyond the individual level. Previous study has reported that exposure to multiple sources of HIV information from mass media was significantly related to HIV knowledge and less stigmatizing attitude towards PLHIV.^49^ Enhancing the content and penetration of HIV/AIDS campaigns within various channels of the media can be an important strategy in disseminating HIV knowledge and reducing HIV‐related discrimination. Health‐care professionals should work with the mass media to provide accurate information about HIV and to avoid a misrepresentation of HIV to the public so as to remove the stigmatizing stereotypes attached to HIV.

There are several limitations of the study that should be noted. First, a convenience sampling was used. Therefore, results cannot be generalized to the whole PLHIV community in Hong Kong. In addition, as PLHIV is a highly protected population, participants were all recruited from HIV‐related NGOs. It might be possible that those who were affiliated with HIV‐related organizations had lower level of stigma, and those who had higher level of stigma were more likely to isolate themselves and less likely to be approached. Similarly participants were self‐selected, and it might be possible that those who chose to participate had a more positive view towards themselves. In addition, the background characteristics of the participants varied greatly in the present study, it might be possible that those with different length of diagnosis, or sexual orientation might have different experiences of stigma. Finally, participants were asked to share their experiences and examples of stigmatization in general, and no attempt has been made to compare their level of stigmatization between different points of time; therefore, there was no information on whether participants’ level of stigmatization has improved over time. These limitations should be considered when interpreting the findings of the study.

The present study described and identified the stigmatizing experiences and possible factors that may lead to stigmatization of PLHIV in Hong Kong. Findings suggest that misconceptions about HIV, together with physical symptoms, attribution of personal responsibility of disease, discrimination and social rejection, and the negative stereotypes of HIV endorsed by the society all contributed to intensifying the level of stigma among PLHIV in Hong Kong. Interventions to reduce stigma of PLHIV are strongly warranted and should target not only the patients themselves, but also their family members, health‐care professionals and the broader community.

## CONFLICT OF INTEREST

All authors declare that they have no conflict of interests.
